# Complete Resection of Simultaneous Hepatic and Pulmonary Metastases from Colorectal Cancer in the Era of Modern Systemic and Surgical Therapies: A Systematic Review and Meta-Analysis

**DOI:** 10.1245/s10434-026-20323-x

**Published:** 2026-08-22

**Authors:** Dominik Wróbel, Patryk Rogaczewski, Patryk Dąbrowski, Wojciech Olejek, Jędrzej Borowczak, Paweł Michał Potocki

**Affiliations:** 1 Jagiellonian University Medical College, Kraków, Poland; 2 Faculty of Medicine, Bydgoszcz University of Science and Technology, Bydgoszcz, Poland; 3 Clinical Department of Oncology, Oncology Centre, Prof. Franciszek Łukaszczyk Memorial Hospital, Bydgoszcz, Poland; 4 Department of Clinical Oncology, Jagiellonian University Medical College, Kraków, Poland

**Keywords:** Colorectal cancer, Liver metastasis, Pulmonary metastasis, Metastasectomy, Overall survival

## Abstract

**Background:**

Data suggest promising outcomes with resection of simultaneous hepato-pulmonary metastases (HPM) from metastatic colorectal cancer (mCRC). However, there is no consensus regarding the effectiveness of this approach. Therefore, we aimed to evaluate the feasibility and clinical benefit of metastasectomy of simultaneous HPM from mCRC.

**Methods:**

A systematic review and meta-analysis were conducted in accordance with Preferred Reporting Items for Systematic Reviews and Meta-Analyses (PRISMA) guidelines, including studies from PubMed and Embase up to October 1, 2025. Outcomes were reported as proportions and as hazard ratios (HRs) with 95% confidence intervals (CIs).

**Results:**

In total, 11 studies, encompassing 507 patients with HPM who underwent complete resection, were included. The 5-year overall survival (OS) rate after metastasectomy was 53.61% (95% CI 41.44–65.79), whereas the 3-year OS rate was 70.9% (95% CI 61.73–80.06). The 5-, 3-, and 1-year disease-free survival rates were 14.73%, 19.48%, and 55.31%, respectively. Complete resection was achieved in 45.65% of patients with initial curative intent, with disease progression accounting for 93.18% of treatment failures. Complete metastasectomy was associated with improved OS compared with isolated liver metastasectomy with systemic treatment of pulmonary metastases (HR 0.30; 95% CI 0.19–0.45, p<0.001), whereas isolated liver resection also improved OS compared with systemic treatment alone (HR 0.37; 95% CI 0.27–0.51, p<0.001).

**Conclusion:**

In the era of modern therapies, complete resection of HPM from mCRC is associated with a substantial survival benefit and should be considered a primary treatment goal in selected patients. However, less than half of patients with initial curative intent complete the planned treatment. Isolated liver metastasectomy may still confer a survival benefit compared with systemic treatment alone.

**Supplementary Information:**

The online version contains supplementary material available at https://doi.org/10.1245/s10434-026-20323-x.

Colorectal cancer is the third most diagnosed malignancy globally and ranks as the second leading cause of cancer-related mortality, primarily due to distant metastatic spread.^1^ The liver and lungs are the most frequent sites of metastasis, with hepatic metastases (HM) occurring in approximately 50% of patients and pulmonary metastases (PM) in up to 20%.^2^ Despite recent advances in systemic therapy, outcomes in metastatic colorectal cancer (mCRC) remain suboptimal. Consequently, the limited efficacy of systemic treatment continues to drive the exploration of metastasectomy in rigorously selected patients, with accumulating evidence supporting its therapeutic value, even when combined with modern chemotherapy regimens.^3^

Although substantial evidence supports the resection of isolated liver metastases, clinical evidence and standardized treatment algorithms remain limited for simultaneous hepato-pulmonary metastases (HPM), which occur in 3.4% of patients diagnosed with CRC^4^ The available literature provides heterogeneous yet predominantly favorable data, supporting the feasibility and potential efficacy of resection in selected patients.^5–15^ In line with these findings, complete metastasectomy is generally recommended when metastases are deemed resectable according to the guidelines of the European Society for Medical Oncology, National Comprehensive Cancer Network, or the Japanese Society for Cancer of the Colon and Rectum. ^16–18^ Nonetheless, these studies vary substantially in patient demographics and surgical or peri-surgical approaches, necessitating quantitative and qualitative syntheses to provide more comprehensive and definitive evidence on metastasectomy for simultaneous HPM.

Therefore, this meta-analysis primarily aims to evaluate the feasibility and efficacy of complete metastasectomy in patients with simultaneous HPM from mCRC. Furthermore, since resection of HM combined with conservative management of PM has shown promising results in some studies, we compared survival between partial metastasectomy, complete metastasectomy, and systemic treatment alone.^5^

## Methods

This study was performed in accordance with the Preferred Reporting Items for Systematic Reviews and Meta-Analyses (PRISMA) statement. The study protocol was registered in the PROSPERO database (ID: CRD420251117480).

### Data Sources and Literature Searches

The PubMed and Embase databases were systematically searched from January 2000 to September 1, 2025. The search strategy combined terms including "metastasectomy", "colorectal cancer", "hepatic", "pulmonary", and “metastases”. The full search strategy is detailed in Supplementary Table 1. Only articles published in English were considered eligible. Additionally, two investigators (D.W. and P.R.) performed independent manual searches to identify relevant studies not captured in the database search (Supplementary Table 1).

### Eligibility Criteria

Eligible studies included patients diagnosed with metastatic colorectal cancer with simultaneously detected liver and lung metastases who underwent complete metastasectomy, defined as radical resection of all metastases at both sites. Additionally, if a small number of patients in a particular study underwent curative treatment other than resection, and the great majority underwent resection, those studies were also allowed. To ensure contemporariness of the surgical procedures and chemotherapy regimens, only studies published after 2000 were included. Furthermore, only studies comprising more than 10 participants with the outcome of interest were deemed eligible. The definition of simultaneous detection varied across studies, with most authors defining it as metastases detected at the same time or within 3 months of the detection of metastatic disease. The exact thresholds are listed in Table 1.

**Table 1:** Baseline characteristics of included studies

Study, country	Time for sim detection	Pts with complete resection	Pt age, years	Female	Pts receiving perioperative chemotherapy	Chemotherapy regimen	Days between hepatic and pulmonary resections, days
Mise et al.^5^; USA	Sim	41	57 (33–73)	15 (37)	38 (92) preop	Oxaliplatin and/or irinotecan (83); bevacizumab (25); cetuximab or panitumumab (18)	NA
Tanaka et al.^6^; Japan	Sim	14	66 (48–78)	7 (50)	8 (52) postop	5-FU (52)	71
Dave et al.^8^; UK	Sim	37	64.2 ± 1.6	8 (21.62)	8 (21.62) after hepatectomy	NA	180 ± 24.4
Andres et al.^9^; Switzerland, France, Russia, Portugal, Spain, Belgium	3 mo	146	60.3 ± 10.8	56 (38.37)	82 (56.16^a^) before hepatectomy; 71 (47.65^a^) after hepatectomy	Preop: 5-FU 83.33^a^, oxaliplatin 62.34^a^, irinotecan 29.63^a^ (percentage of preop regimens) Postop: 5-FU 81.25^a^, oxaliplatin 54.69^a^, irinotecan 28.13^a^ (percentage of postop regimens)	NA
Matsumura et al.^10^; Japan	Sim	33	64 (35–83)	17 (51.5)	12 (36.4) preop	Oxaliplatin 21.2, irinotecan 9.1, bevacizumab 21.2, anti-EGFR 0	45 (0–131)
Pulitano et al.^11^; UK, Italy, Austria, USA	Sim	62	NA	NA	NA	NA	NA
Shimizu et al.^12^; Japan	3 mo	28	NA	NA	Pulmonary resection, 0; Liver resection, NA	NA	NA
Mineo et al.^13^; Italy	1 mo	12	NA	4 (33.33)	NA	NA	NA
Engstrand et al.^14^; Sweden	6 mo	39	63 (43–82)	15 (38)	33 (85) preop	NA	NA
De Bellis et al.^15^; Italy	3 mo	63	Transdiaphragmatic 53 (41–56); transthoracic 57 (47–63); staged 59 (43–66)	29 (46.03)	59 (93.65) preop; 35 (55.56) postop	NA	NA
Takeda et al.^7^; Japan	Sim	32	NA	9 (28.2)	NA	NA	63.5

Data are presented as N, N (%), mean ± standard deviation, or median (interquartile range) unless otherwise indicated. 5-FU, 5-fluorouracil; EGFR, epithelial growth factor receptor; IQR, interquartile range; mo, month(s); NA, not available; postop, postoperative; preop, preoperative; pt, patient(s); SD, standard deviation; Sim, simultaneous. ^a^ Values for some patients are missing

### Outcomes of Interest

The primary outcome of interest was the pooled 5-year overall survival (OS) rate with 95% confidence intervals (CIs). Secondary outcomes included the 3-year OS rate, 1-, 3-, and 5-year disease-free survival (DFS) rates, and the proportion of patients who underwent complete metastasectomy at both sites (hepatic and pulmonary) among those initially planned for curative-intent resection. For the primary outcome, subgroup analyses were performed to identify sources of heterogeneity and attempt reduction of thereof. Furthermore, the frequency of reasons for treatment failure were pooled. Finally, we compared OS between patients undergoing partial metastasectomy (hepatic resection with systemic treatment of PM), complete metastasectomy, and systemic treatment alone, using hazard ratios (HRs) with 95% CIs.

### Study Selection

**Fig. 1:**
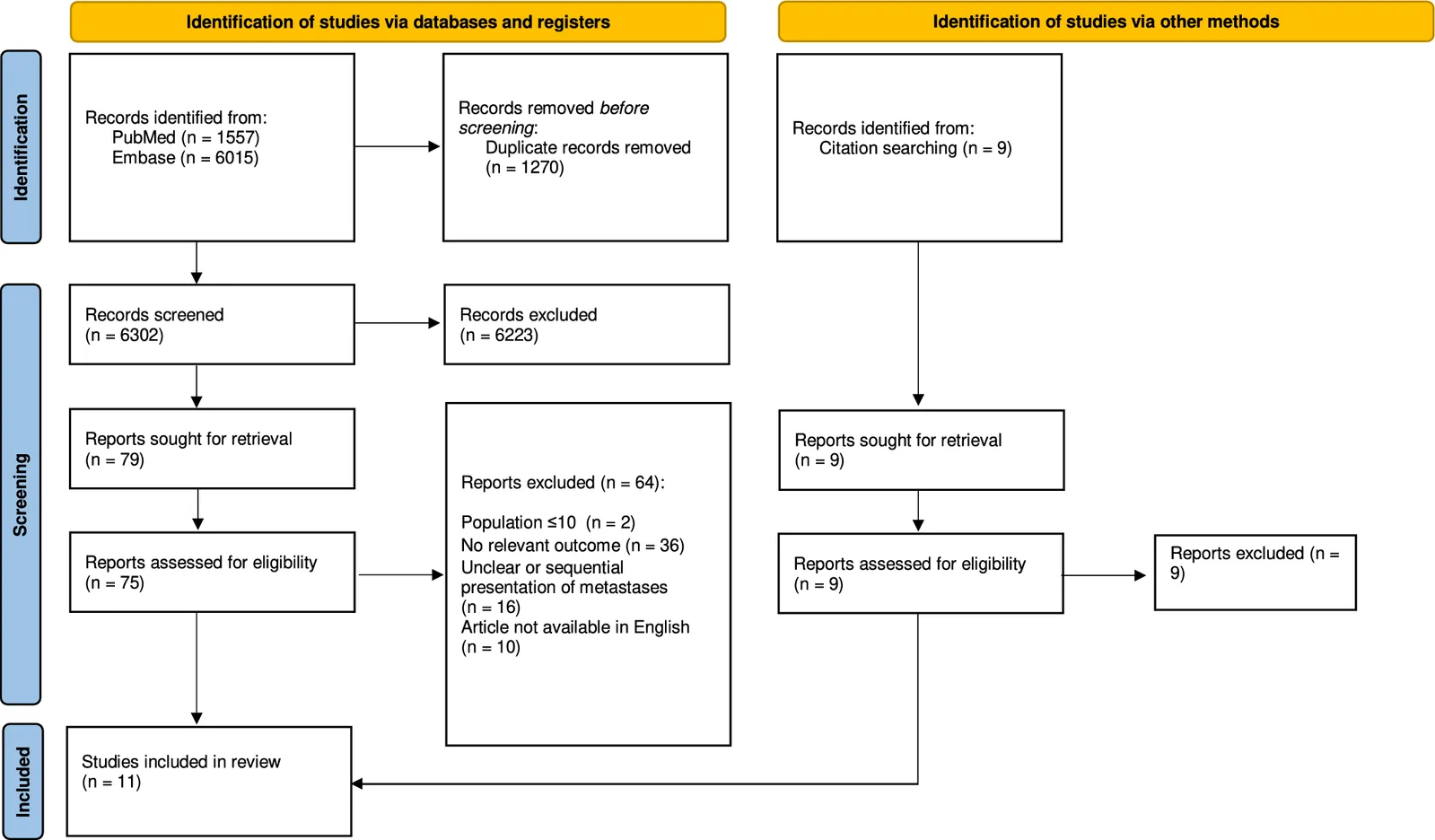
Preferred reporting items for systematic reviews and meta-analyses (PRISMA) flowchart for included studies

Two investigators (W.O. and P.D.) independently screened titles and abstracts for eligibility based on the predefined inclusion and exclusion criteria, followed by a full-text review of potentially relevant studies. Disagreements were resolved by consensus with a third investigator (D.W.). The study selection process is summarized in the PRISMA flow diagram (Fig. 1).

### Data Extraction and Verification

Data were extracted independently by two investigators (W.O. and P.D.) using a predesigned spreadsheet to ensure standardized collection of variables, including authorship, publication year, study region, study design, adjuvant/neoadjuvant treatment, surgical details, follow-up details, sample size, demographics, treatment allocations, and outcomes of interest. Extracted data were cross-verified by a third investigator (D.W.) to ensure accuracy and integrity. The reference lists of all included studies were manually searched to identify additional eligible publications. Any discrepancies in extracted data were resolved by discussion or consensus.

### Quality Assessment

The methodological quality of the included studies was independently assessed by two investigators (D.W. and P.R.), with discrepancies resolved by consensus with a third investigator (P.P.). The Newcastle–Ottawa Quality Assessment Scale (NOS) was selected as the methodological quality assessment tool (Supplementary Fig. 1)^19^.

### Statistical Analysis

Pooled estimates, along with their corresponding 95% CIs were calculated for the respective outcomes. Study heterogeneity was assessed by the *I*^*2*^ statistic, interpreted as low (25–50%), moderate (50–75%), or high (>75%).^20^ A random-effects model using the restricted maximum likelihood method was applied to account for differences in population and study setting.^21^ Publication bias was evaluated visually using funnel plots and statistically using Egger’s regression test; p < 0.05 indicated potential asymmetry. Whenever possible, outcomes directly reported by authors were extracted; in other instances, *IPDfromKM* web-based Shiny application was used to reconstruct individual patient data from published Kaplan–Meier curves and to calculate rates of survival at specific time points.^22^ For studies comparing treatment strategies, if HRs and corresponding 95% CIs were not provided for the comparison of treatment modalities of interest, they were derived using the method proposed by Tierney et al.^23^ All quantitative analyses were performed using STATA version 19.5 (StataCorp LP, College Station, TX, USA).

## Results

Database searches identified 7572 studies, of which 1270 were duplicates. Among the remaining 6302 records, 6223 were excluded after title and abstract screening. A total of 79 articles were analyzed during full-text review, of which 11 met the inclusion criteria (Fig. 1).^5–15^ All included studies were retrospective cohort studies published between 2003 and 2024. In total, the meta-analysis included 507 patients who underwent complete resection of simultaneous HPM. The main characteristics of the included studies are summarized in Table 1, with more detailed clinicopathological data provided in Supplementary Table 2. The results of the methodological quality assessment are shown in Supplementary Fig. 1.

### Overall Survival

**Fig. 2:**
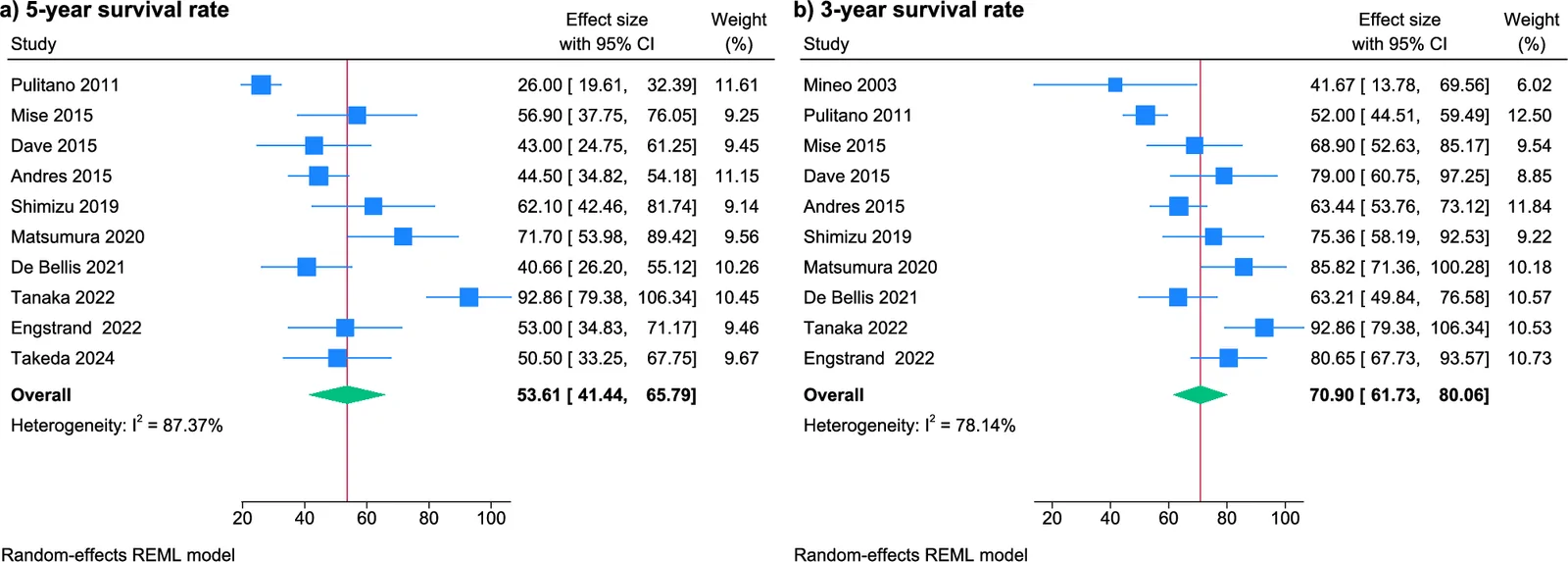
Overall survival rates among patients with complete metastasectomy of hepato-pulmonary metastases from metastatic colorectal cancer. CI, confidence interval. REML, residual maximum likelihood

A total of 10 studies, encompassing 495 patients, were included in the analysis of 5-year OS.^5–12,14,15^ Estimated 5-year OS after successful metastasectomy (most commonly calculated from the time of primary hepatic resection; see Table 1) was 53.61% (95% CI 41.44–65.79), with high heterogeneity across studies (Fig. 2a). For 3-year OS, 10 studies including 475 patients were analyzed.^5,6,8–15^ Estimated 3-year OS was 70.9% (95% CI 61.73–80.06), with high heterogeneity, albeit slightly lower than that observed for 5-year OS (Fig. 2b).

#### Sensitivity Analysis for 5-Year Overall Survival

**Fig. 3:**
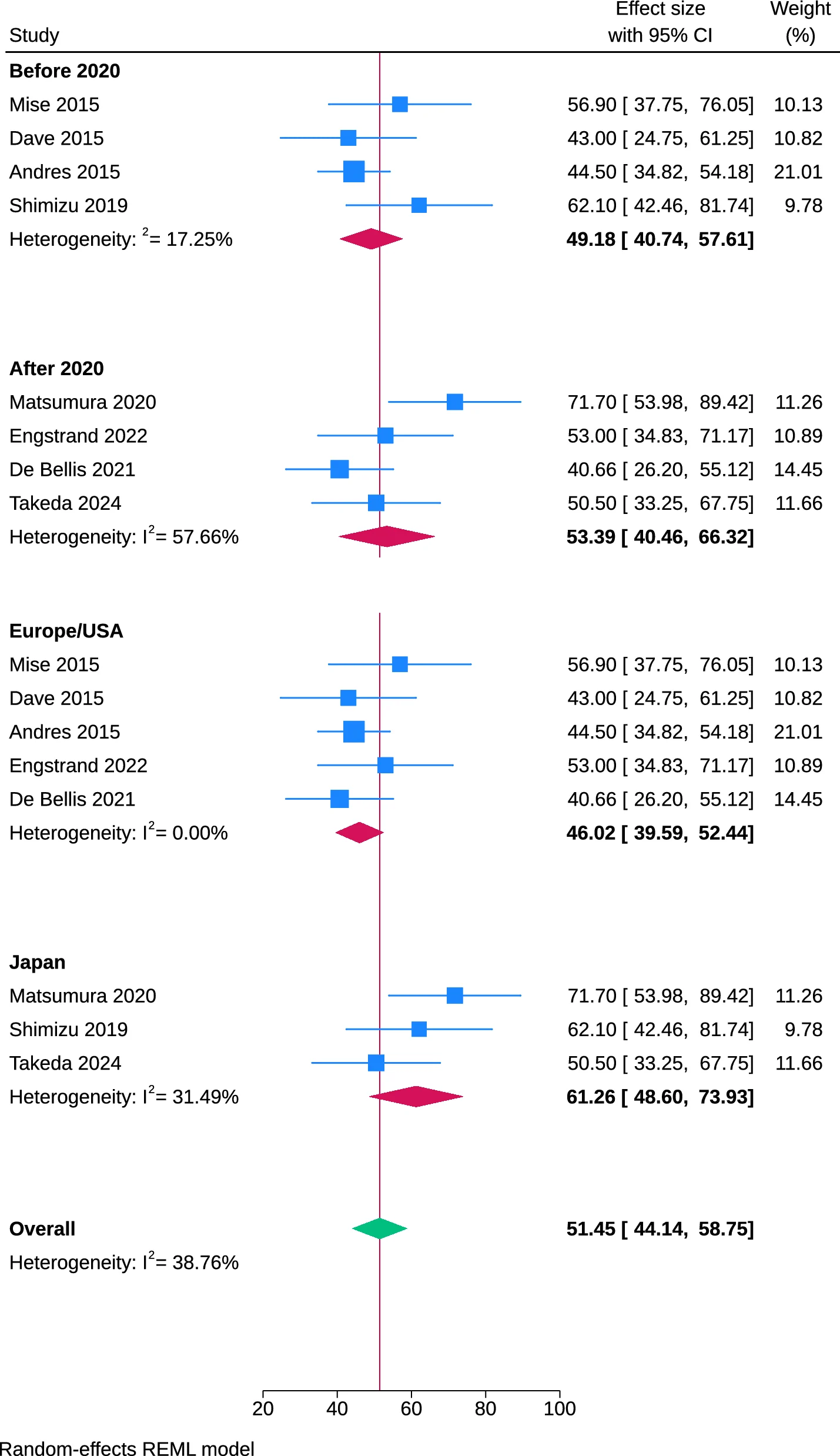
Subgroup analysis performed for 5-year overall survival, according to publication year and study region. CI, confidence interval; REML, residual maximum likelihood

To reduce heterogeneity, we performed an additional analysis of 5-year OS, excluding two outlier studies: one with the lowest and one with the highest 5-year OS estimate. In this analysis, eight studies encompassing 419 patients were included.^5,7–10,12,14,15^ Estimated 5-year OS for this subset was 51.45% (95% CI 44.14–58.75), with substantially reduced heterogeneity compared with the full set of studies (Fig. 3).

#### Subgroup Analysis of 5-Year OS

Subsequently, we conducted subgroup analyses to explore variations in treatment outcomes according to selected confounders. A stronger effect was observed in studies published in or after 2020, with 5-year OS of 53.39% (95% CI 40.46–66.32) compared with 49.18% (95% CI 40.74–57.61) in studies published before 2020 (Fig. 3). A stronger effect was also observed in Japanese studies, with 5-year OS of 61.26% (95% CI 48.60–73.93), compared with 46.02% (95% CI 39.59–52.44) in studies from the USA or Europe.

### Disease-Free Survival

**Fig. 4:**
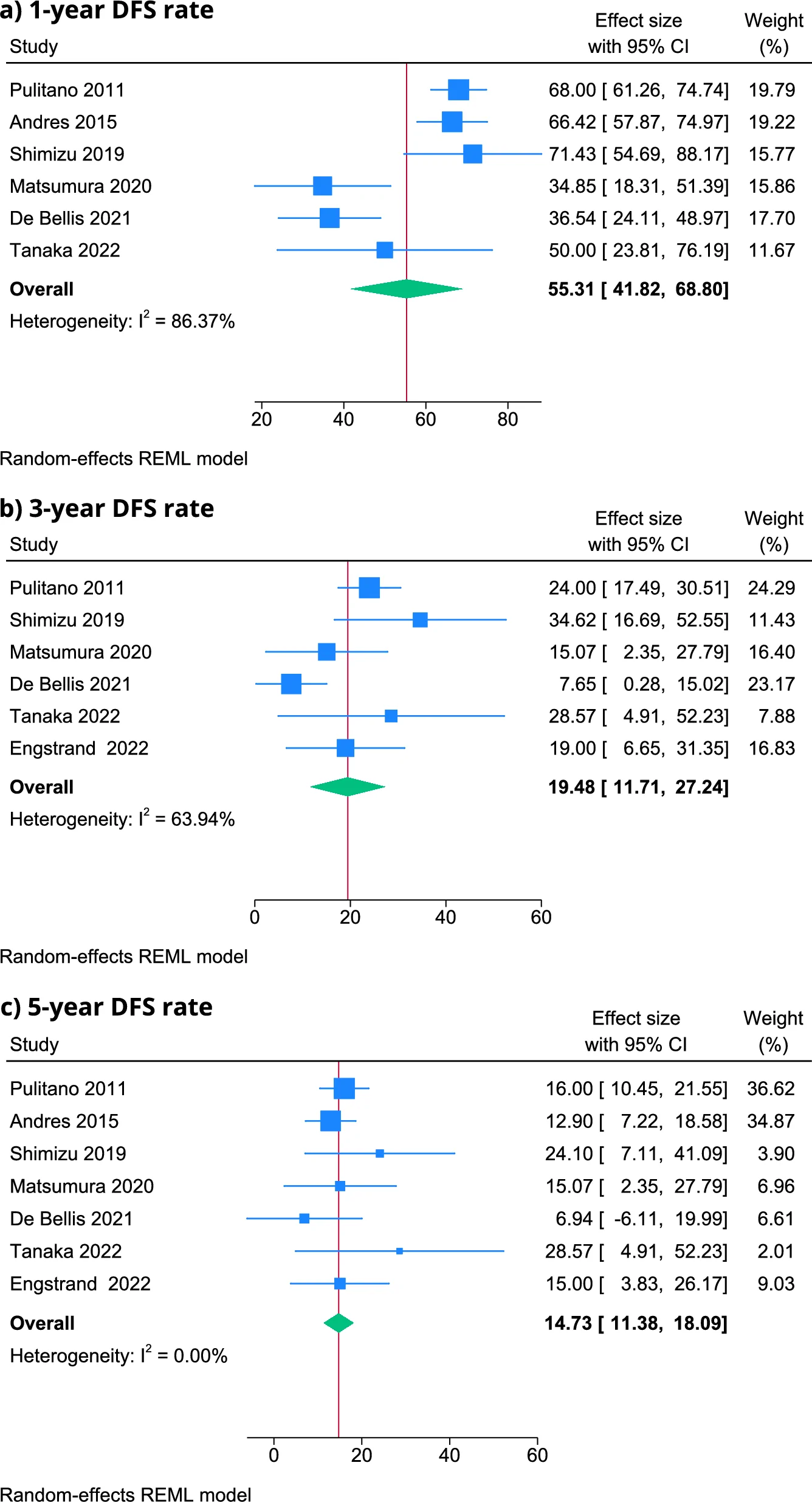
**a** 1-year, **b** 3-year, and **c** 5-year disease-free survival among patients with complete metastasectomy. CI, confidence interval; REML, residual maximum likelihood

A total of seven studies, including 385 patients, were analyzed for 5-year DFS, which was 14.73% (95% CI 11.38–18.09), with no heterogeneity (Fig. 4c).^6,9–12,14,15^ Furthermore, six studies, including 239 patients, were analyzed for 1- and 3-year DFS, which were 55.31% (95% CI 41.82–68.8) and 19.48% (95% CI 11.71–27.24), with high and moderate heterogeneity, respectively (Fig. 4a, 4b).^6,10–12,14,15^

### Complete Resection Rate

**Fig. 5:**
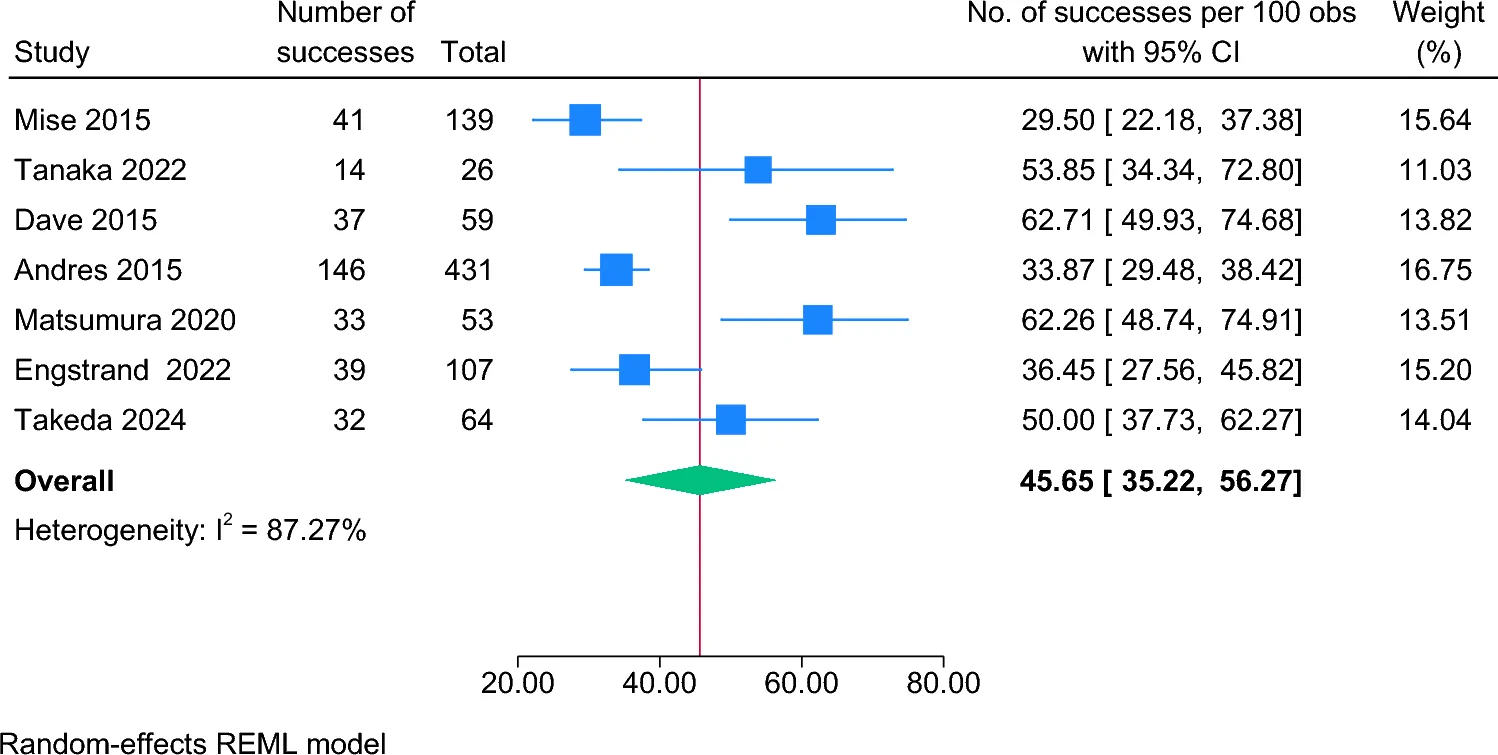
Complete resection rate among patients with patient with initial curative intent. CI, confidence interval; REML, residual maximum likelihood

To determine the proportion of patients initially deemed resectable at both sites who ultimately underwent complete resection, we analyzed the complete resection rate. A total of seven studies including 879 patients who underwent hepatic metastasectomy and were resected were included in the analysis.^5–10,14^ The pooled complete resection rate (hepatic followed by pulmonary resection) was 45.65% (95% CI 35.22–56.27), with high heterogeneity (Fig. 5).

We also analyzed the causes of failure to achieve complete resection in patients initially planned to receive curative-intent treatment. Based on five studies including 230 patients who did not undergo complete resection, the main causes were disease progression or recurrence (93.18%; 95% CI 84.57–98.81), complications (1.76%; 95% CI 0–7.93), treatment refusal (1.54%; 95% CI 0–4.97), and patient deterioration (1.01%; 95% CI 0–4.91) (Supplementary Fig. 2).^5–7,10,14^

### Repeated Resection Rate

As most patients eventually experience recurrence, even after complete resection, we analyzed the proportion of patients who underwent repeat resections for recurrence after an initially complete resection. Based on two studies, including 60 patients with recurrence after complete resection, the repeated resection rate was 60.03% (95% CI 47.13–72.29), with no heterogeneity (Supplementary Fig. 3).^10,14^

### Outcomes of Partial Resection

To evaluate whether isolated resection of liver metastases with systemic treatment of PM constitutes a viable option, we compared OS between patients undergoing complete resection at both sites, isolated liver metastasectomy with systemic treatment, and systemic treatment with no resection at either site.

**Fig. 6:**
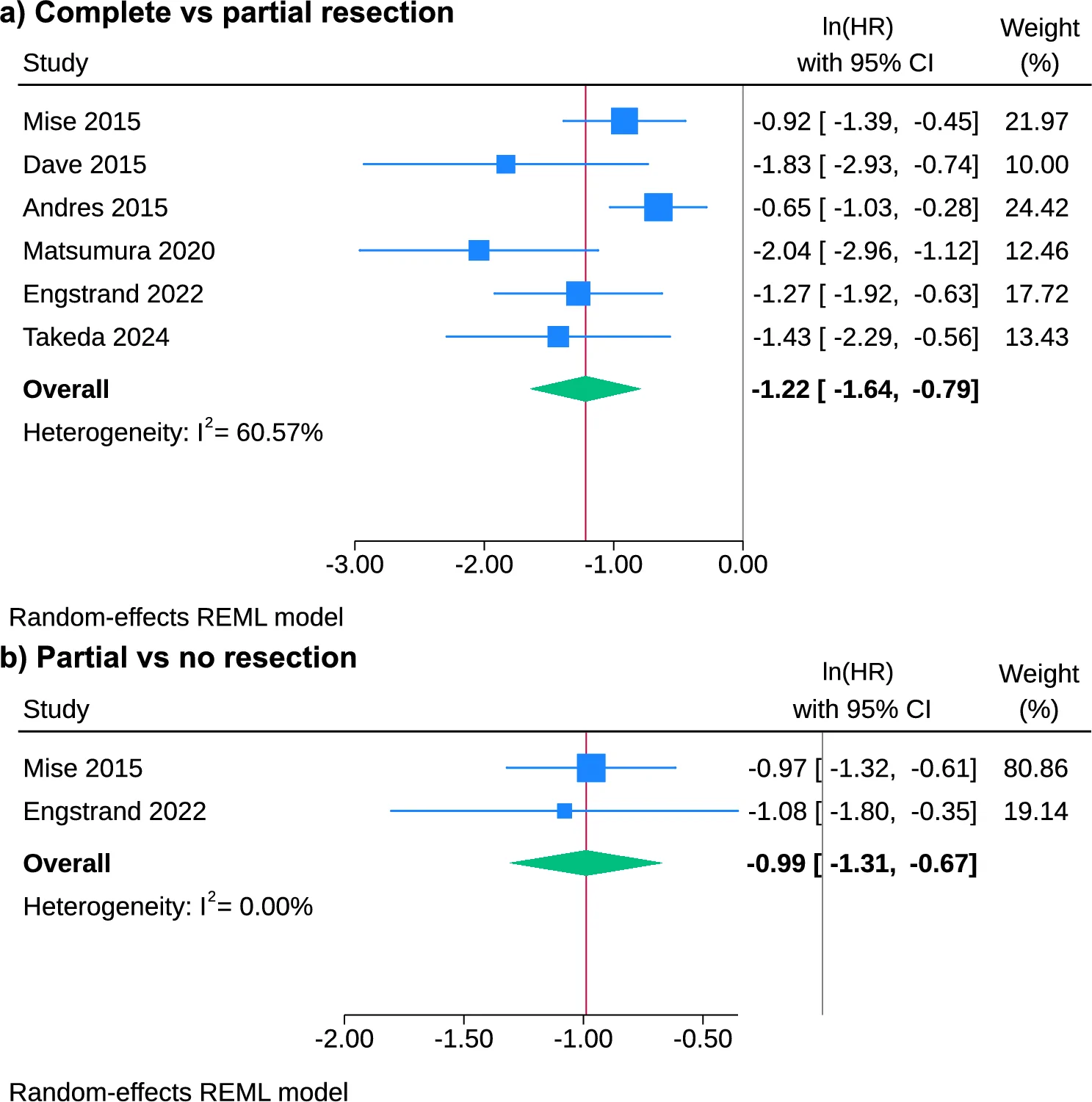
Overall survival between groups with **a** complete vs partial resection and **b** partial vs no resection. CI, confidence interval; REML, residual maximum likelihood

A total of six studies, including 812 patients, were included in the analysis comparing complete resection at both sites with isolated liver metastasectomy and systemic treatment.^5–10^ Complete resection was associated with improved OS (HR 0.30; 95% CI 0.19–0.45; p < 0.001), with moderate heterogeneity. Furthermore, based on two studies including 162 patients, partial resection was associated with improved OS compared with systemic therapy only (HR 0.37; 95% CI 0.27–0.51, p < 0.001), with no heterogeneity (Fig. 6).^5,14^

### Publication Bias

Analysis of publication bias for the main outcome (5-year OS) suggested a low risk of bias (Egger’s p = 0.1959) (Supplementary Fig. 4).

## Discussion

To the best of our knowledge, this is the first meta-analysis focusing specifically on long-term survival after metastasectomy of simultaneous HPM from CRC. The results suggest that, in carefully selected patients, complete resection at both sites yields a 53.61% survival rate at 5 years following the initial metastasectomy, decisively outperforming outcomes of contemporary systemic therapies. These results support the pursuit of surgical treatment in carefully selected patients, especially in highly specialized centers.

The survival rate reported in our study is comparable to that observed after resection of liver metastases.^24^ It is considerably higher than that in a recently published large population-based study, in which 3-year survival was 29%, and in a similarly scoped systematic review reporting 5-year survival of 27%^3,25^ These discrepancies can be at least partially explained by differences in methodology. First, for the primary endpoint analysis, we included only patients with complete curative-intent resection, whereas Siebenhüner et al.^25^ included all patients who underwent metastasectomy, including those with palliative, incomplete, and non-synchronous resections, with limited details on patient pathways. Second, in their analysis, survival rates appear markedly lower than expected based on modern treatment approaches. Notably, for liver metastasis resection, the reported 3-year survival rates correspond to those of the highest-risk prognostic group in the classification proposed by Buisman et al.^24^ Furthermore, a previous systematic review applied less strict inclusion criteria and was published nearly 15 years ago.^3^ Given the significant improvement in systemic treatment and surgical techniques, this discrepancy can likely be attributed to such progress. As such, we believe patient selection is key to achieving long-term survival in this setting and that the results of our study should be primarily applied to these carefully selected patients treated in highly specialized centers.

Another factor limiting the broader applicability of our results is the relatively high heterogeneity observed for 5-year survival. However, this was at least partially mitigated by excluding studies with the most outlying results and performing subgroup analyses. As expected, outcomes were better in studies published in or after 2020 than in those published earlier, although the difference was modest. Interestingly, when stratified by region, Japanese studies showed better survival outcomes than those conducted in the USA and Europe. We suspect that this may be related to higher rates of repeat resection reported in Japanese cohorts, which may prolong survival.^26–28^ Unfortunately, direct comparison was not possible, as only two studies reported data on repeat resections.

The results demonstrated that, although more than half of patients undergoing complete metastasectomy survive 5 years, disease recurrence occurs in up to 85% by that time. Nearly half of these recurrences are diagnosed within the first year after the initial liver metastasectomy, with a further 35% occurring within the next 2 years and only 5% between 3 and 5 years. These findings provide valuable clinical insight into the expected timing of recurrence after “complete” resection, although they do not account for other important factors such as carcinoembryonic antigen levels or RAS/RAF mutation status. Furthermore, they imply that, despite a clear survival benefit in selected patients, complete metastasectomy rarely achieves a cure.

This discrepancy between relatively poor DFS and favorable OS may seem counterintuitive but can be at least partially explained by the use of post-recurrence treatments. In many cases, recurrences after metastasectomy remain amenable to further surgical treatment, provided they are detected early and the patient is fit for repeat surgery. Although only two studies reported repeated resections after completion of treatment, they were performed in 60.03% of patients, suggesting that this approach is a feasible option in a substantial proportion of cases. Moreover, Matsumura et al.^10^ showed that time to surgical failure – defined as the duration from initial resection to the first unresectable recurrence – was more strongly associated with OS than DFS itself. This finding underscores the importance of intensive follow-up even after complete metastasectomy, as it may allow for further curative-intent resection or timely initiation of systemic therapy or radiotherapy.

We demonstrated that only 45.65% of patients undergoing curative-intent treatment completed the initial treatment plan. Although pooling the reasons for failure was challenging and feasible in only five studies, the vast majority of failures were due to disease progression or recurrence (93.18%), followed by complications, clinical deterioration, and patient refusal of further invasive treatment. These results help set realistic expectations for both physicians and patients. Given that more than half of patients may not complete the planned lung resection, simultaneous resection of metastases at both sites should be considered when feasible. Engstrand et al.^14^ showed that, although simultaneous resection is technically challenging, it was associated with a higher rate of complete curative resection than the liver-first approach.

Another putative approach for patients with simultaneous HPM is to resect liver metastases while leaving PM untreated surgically and instead managing them with systemic therapy. This strategy is based on the rationale that lung metastases are generally smaller than those in the liver and may be more efficiently controlled with chemotherapy, as suggested in some studies.^5^ Accordingly, our analysis showed that patients undergoing liver resection combined with systemic treatment for PM had improved OS compared with those receiving systemic treatment alone at both sites. However, complete resection remained associated with significantly improved OS compared with partial resection across all included studies.

Therefore, these results position complete metastasectomy as the primary treatment goal in patients with simultaneous HPM, while also providing a rationale for hepatic resection even when PM are unresectable or lung resection is not feasible. An alternative approach is to treat PM with radiotherapy, such as stereotactic body radiotherapy (SBRT), which has also shown efficacy in this setting.^6^ Unfortunately, the effectiveness of SBRT could not be comprehensively evaluated in this analysis.

This study presents several important limitations. First, and most importantly, the long inclusion period spans substantial changes in diagnostic and treatment modalities, including earlier detection of metastases, improved systemic treatment, and advances in surgical techniques. This temporal effect was reflected in the subgroup analysis, with better survival observed in studies published after 2020 than in those published earlier. Second, there was considerable heterogeneity among studies in terms of perioperative chemotherapy regimens, surgical approaches, follow-up strategies, and resection eligibility criteria, with some studies lacking detailed reporting, thereby limiting the generalizability of our results. Third, definitions of “simultaneous” HPM varied across studies: six defined it as occurring at the same time, one within 1 month, three within 3 months, and one within 6 months. Although subgroup analysis did not reveal meaningful survival differences across these definitions, patients presenting truly simultaneously or within a shorter interval may represent a more aggressive disease phenotype. Finally, a small number of patients underwent SBRT or thermal ablation instead of surgical resection in the studies by Engstrand et al.^14^ and Dave et al.^8^ (13 and 4 patients, respectively). Given their limited number and likely marginal impact on the overall results, these studies were not excluded from the analysis.

## Conclusion

This study strongly supports complete resection of simultaneous HPM whenever feasible, as more than half of these patients survive 5 years when resection is achieved. At the same time, less than half of patients with initial curative intent complete the planned treatment, highlighting the need for realistic expectations among both physicians and patients. Further studies are needed to identify predictors of complete resection and to establish standardized treatment algorithms.

## Supplementary Information

Below is the link to the electronic supplementary material.

Supplementary file 1: Fig.1 Risk of bias assessment according to the Newcastle-Ottawa Quality Assessment Scale

Supplementary file 2: Fig. 2 Reasons for treatment failure in patients with initially curative intent

Supplementary file 3: Fig.3 Repeated resection rate among patients with complete metastasectomy

Supplementary file 4: Fig.4 Funnel plot for publication bias assessment

Supplementary file 5

Supplementary file 6: Table 1 Search strategy

## Data Availability

Data are provided within the manuscript.
