# Supplementary material for: Complete Resection of Simultaneous Hepatic and Pulmonary Metastases from Colorectal Cancer in the Era of Modern Systemic and Surgical Therapies: A Systematic Review and Meta-Analysis
Source: Ann Surg Oncol. 2026 Aug 22;33(12):10065–75. doi: 10.1245/s10434-026-20323-x (PMC13675156; doi:10.1245/s10434-026-20323-x)
Supplement: Supplementary file 1 [file 10434_2026_20323_MOESM1_ESM.pdf]

NOS Scores by Study

Author, Year	Selection	Comparability	Outcome/Exposure	Total Score	Overall RoB
De Bellis, 2021	2	0	3	5	Moderate
Pulitano, 2011	3	0	2	5	Moderate
Shimizu, 2019	2	0	3	5	Moderate
Tanaka, 2022	4	0	2	6	Moderate
Andres, 2015	3	2	2	7	Low
Dave, 2015	4	0	3	7	Low
Engstrand, 2022	4	0	3	7	Low
Matsumura, 2020	4	0	3	7	Low
Mise, 2015	4	0	3	7	Low
Takeda, 2024	4	0	3	7	Low
Mineo, 2003	2	0	1	3	High

Overall Risk
- High
- Moderate
- Low
