# Supplementary material for: Complete Resection of Simultaneous Hepatic and Pulmonary Metastases from Colorectal Cancer in the Era of Modern Systemic and Surgical Therapies: A Systematic Review and Meta-Analysis
Source: Ann Surg Oncol. 2026 Aug 22;33(12):10065–75. doi: 10.1245/s10434-026-20323-x (PMC13675156; doi:10.1245/s10434-026-20323-x)
Supplement: Supplementary file 2 [file 10434_2026_20323_MOESM2_ESM.pdf]

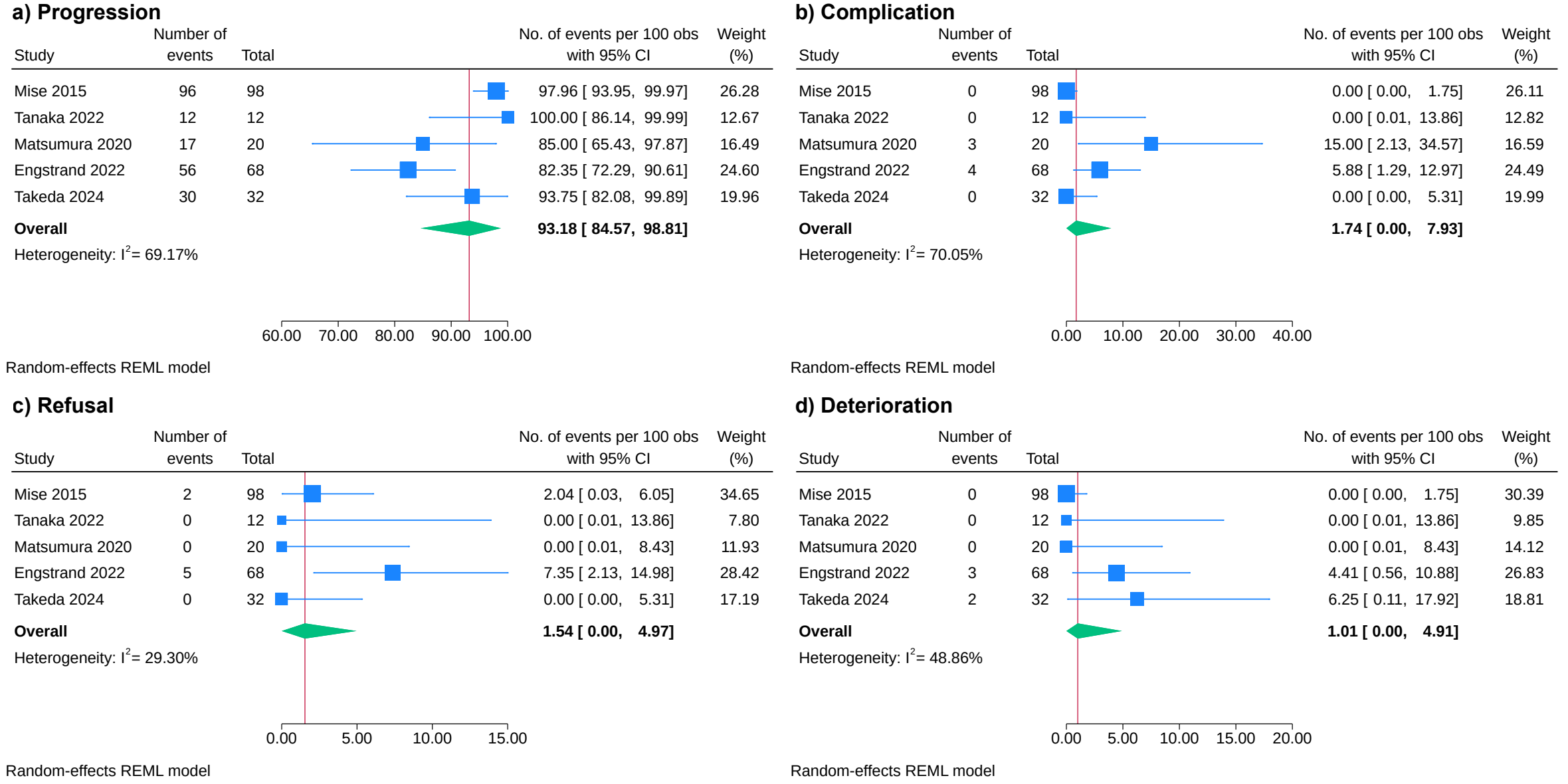
a) Progression
Study | Number of events | Total | No. of events per 100 obs with 95% CI | Weight (%)
Mise 2015 | 96 | 98 | 97.96 [93.95, 99.97] | 26.28
Tanaka 2022 | 12 | 12 | 100.00 [86.14, 99.99] | 12.67
Matsumura 2020 | 17 | 20 | 85.00 [65.43, 97.87] | 16.49
Engstrand 2022 | 56 | 68 | 82.35 [72.29, 90.61] | 24.60
Takeda 2024 | 30 | 32 | 93.75 [82.08, 99.89] | 19.96
Overall | 93.18 [84.57, 98.81]
Heterogeneity: I^2= 69.17%
60.00 70.00 80.00 90.00 100.00
Random-effects REML model
b) Complication
Study | Number of events | Total | No. of events per 100 obs with 95% CI | Weight (%)
Mise 2015 | 0 | 98 | 0.00 [0.00, 1.75] | 26.11
Tanaka 2022 | 0 | 12 | 0.00 [0.01, 13.86] | 12.82
Matsumura 2020 | 3 | 20 | 15.00 [2.13, 34.57] | 16.59
Engstrand 2022 | 4 | 68 | 5.88 [1.29, 12.97] | 24.49
Takeda 2024 | 0 | 32 | 0.00 [0.00, 5.31] | 19.99
Overall | 1.74 [0.00, 7.93]
Heterogeneity: I^2= 70.05%
0.00 10.00 20.00 30.00 40.00
Random-effects REML model
c) Refusal
Study | Number of events | Total | No. of events per 100 obs with 95% CI | Weight (%)
Mise 2015 | 2 | 98 | 2.04 [0.03, 6.05] | 34.65
Tanaka 2022 | 0 | 12 | 0.00 [0.01, 13.86] | 7.80
Matsumura 2020 | 0 | 20 | 0.00 [0.01, 8.43] | 11.93
Engstrand 2022 | 5 | 68 | 7.35 [2.13, 14.98] | 28.42
Takeda 2024 | 0 | 32 | 0.00 [0.00, 5.31] | 17.19
Overall | 1.54 [0.00, 4.97]
Heterogeneity: I^2= 29.30%
0.00 5.00 10.00 15.00
Random-effects REML model
d) Deterioration
Study | Number of events | Total | No. of events per 100 obs with 95% CI | Weight (%)
Mise 2015 | 0 | 98 | 0.00 [0.00, 1.75] | 30.39
Tanaka 2022 | 0 | 12 | 0.00 [0.01, 13.86] | 9.85
Matsumura 2020 | 0 | 20 | 0.00 [0.01, 8.43] | 14.12
Engstrand 2022 | 3 | 68 | 4.41 [0.56, 10.88] | 26.83
Takeda 2024 | 2 | 32 | 6.25 [0.11, 17.92] | 18.81
Overall | 1.01 [0.00, 4.91]
Heterogeneity: I^2= 48.86%
0.00 5.00 10.00 15.00 20.00
Random-effects REML model
