# Supplementary material for: Complete Resection of Simultaneous Hepatic and Pulmonary Metastases from Colorectal Cancer in the Era of Modern Systemic and Surgical Therapies: A Systematic Review and Meta-Analysis
Source: Ann Surg Oncol. 2026 Aug 22;33(12):10065–75. doi: 10.1245/s10434-026-20323-x (PMC13675156; doi:10.1245/s10434-026-20323-x)
Supplement: Supplementary file 3 [file 10434_2026_20323_MOESM3_ESM.pdf]

Study	Number of successes	Total	No. of successes per 100 obs with 95% CI	Weight (%)
Matsumura 2020	18	28	64.29 [45.48, 81.18]	46.72
Engstrand 2022	18	32	56.25 [38.64, 73.13]	53.28
**Overall**			**60.03 [47.13, 72.29]**	

Heterogeneity: $I^2 = 0.00\%$

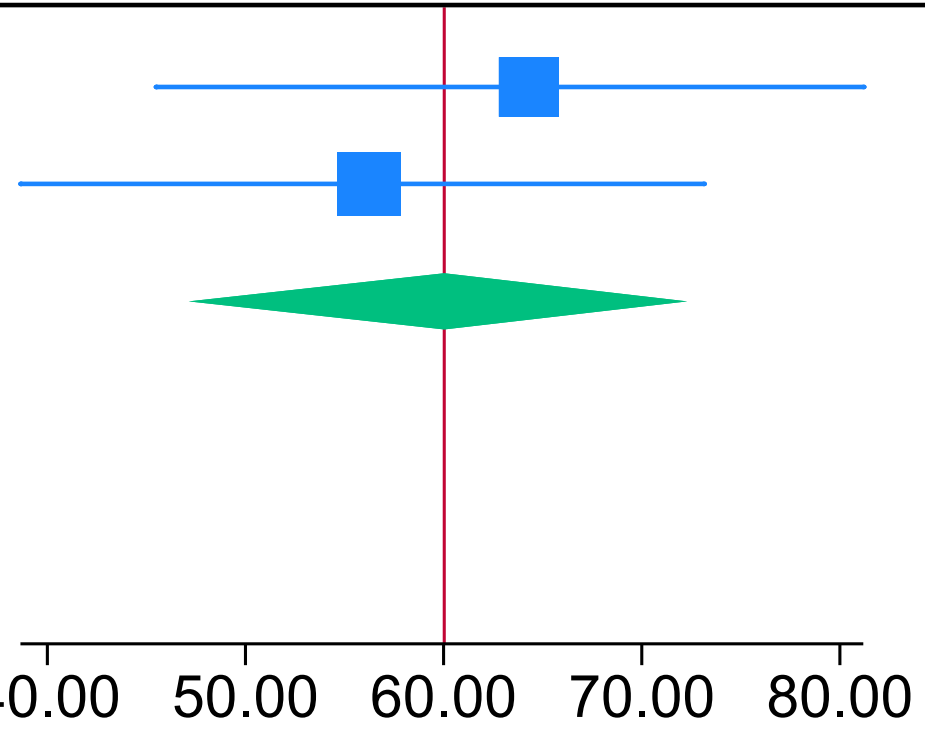

Random-effects REML model
