# Supplementary material for: Complete Resection of Simultaneous Hepatic and Pulmonary Metastases from Colorectal Cancer in the Era of Modern Systemic and Surgical Therapies: A Systematic Review and Meta-Analysis
Source: Ann Surg Oncol. 2026 Aug 22;33(12):10065–75. doi: 10.1245/s10434-026-20323-x (PMC13675156; doi:10.1245/s10434-026-20323-x)
Supplement: Supplementary file 4 [file 10434_2026_20323_MOESM4_ESM.pdf]

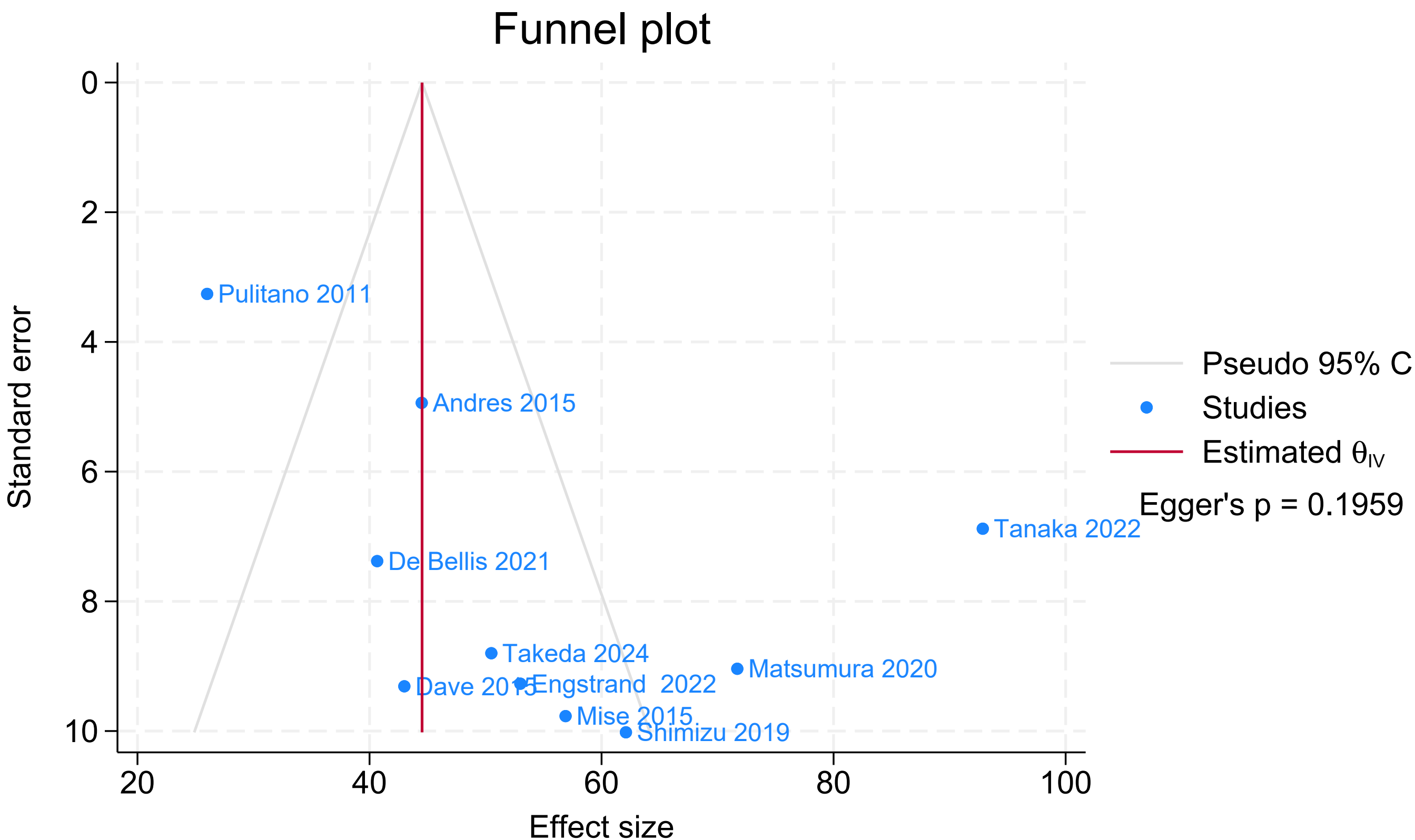

Funnel plot
Standard error
0
2
4
6
8
10
20
40
60
80
100
Effect size
Pulitano 2011
Andres 2015
De Bellis 2021
Tanaka 2022
Takeda 2024
Matsumura 2020
Dave 2015
Engstrand 2022
Mise 2015
Shimizu 2019
Pseudo 95% CI
Studies
Estimated θIV
Egger's p = 0.1959
