# Supplementary material for: Complete Resection of Simultaneous Hepatic and Pulmonary Metastases from Colorectal Cancer in the Era of Modern Systemic and Surgical Therapies: A Systematic Review and Meta-Analysis
Source: Ann Surg Oncol. 2026 Aug 22;33(12):10065–75. doi: 10.1245/s10434-026-20323-x (PMC13675156; doi:10.1245/s10434-026-20323-x)
Supplement: Supplementary file 5 [file 10434_2026_20323_MOESM5_ESM.docx]

**Supplementary Table 2** Detailed clinico-pathological characteristics of the included studies.

| **Study** | **Primary disease in rectum,**  ***N* (%)** | **KRAS mutation, *N* (%)** | **Median/**  **mean number of liver metastases,**  ***N* (IQR or SD)** | **Median/**  **mean**  **diameter of the largest liver metastasis, *cm* (IQR or SD)** | **Bilobar**  **hepatic disease,**  ***N* (%)** | **Median/**  **mean number of lung metastases,**  ***N* (IQR or SD)** | **Median/**  **mean diameter of the largest lung metastasis, *cm* (IQR or SD)** | **Median/**  **mean preoperative CEA level,**  ***ng/mL* (IQR or SD)** | **Bilateral pulmonary disease,**  ***N* (%)** |
| --- | --- | --- | --- | --- | --- | --- | --- | --- | --- |
| Mise 2015 | 8 (20) | N/A | 1 (1–9) | 2.0 (0.5–10.0) | N/A | 2 (1–11) | 13 (3–50) | 2.4 (0.7–383) | 8 (20) |
| Tanaka 2022 | 8 (57.1) | N/A | 2.5 (1–13) | 2.8 (0.8–9) | 7 (50.0) | 2 (1–5) | 0.9 (0.6–2.5) | 6.0 (1.2–502.6) | 5 (35.7) |
| Dave 2015 | N/A | N/A | 3.0 (0.5) | 3.5 (0.54) | 8 (21.62) | N/A | N/A | 86.6 (49.3) | N/A |
| Andres 2015 | 59 (40.69%*) | N/A | N/A | N/A | 38  (25.68%*) | N/A | N/A | N/A | 27  (19.01%*) |
| Matsumura 2020 | 19 (57.6%) | 8 (50%*) | 2 (1-25) | 3.3 (0.3-11) | N/A | 1 (1-5) | 1.1 (0.3-3) | 19.9 (1.5-333) | 8 (24%) |
| Pulitano 2011 | N/A | N/A | N/A | N/A | N/A | N/A | N/A | N/A | N/A |
| Shimizu 2019 | N/A | N/A | N/A | N/A | N/A | N/A | N/A | N/A | N/A |
| Mineo 2003 | 3 (25%) | N/A | 2.1 | N/A | N/A | 2 | N/A | N/A | N/A |
| Engstrand 2022 | 19 (49%) | 10 (53%*) | 2 (1-11) | 2.5 (0.7-10) | N/A | 1 (1-7) | 0.1 (0.4-0.75) | 5.7 (0.5- 138) | 5 (13%) |
| De Bellis 2021 | 26 (41.27%) | 26 (72.22%*) | Transdia-phragmatic  2 (1–3)  Transthoracic  1 (1–2)  Staged  3 (2–3) | Transdia-phragmatic  2.4 (1.5–3.5)  Transthoracic  1.6 (1.4–2.3)  Staged  2 (1.5–3.1) | N/A | Transdia-phragmatic  1 (1–1)  Transthoracic  1 (1–1)  Staged  1 (1–2) | Transdia-phragmatic  0.8 (0.6–1.5)  Transthoracic  1 (0.7–1.8)  Staged  1.2 (1–1.9) | Transdia-phragmatic  4.6 (2.5–17.3)  Transthoracic  3.7 (2–10)  Staged  4 (1.6–13.7) | 5 (8%) |
| Takeda 2024 | N/A | N/A | N/A | N/A | N/A | N/A | N/A | N/A | N/A |

*KRAS - Kirsten rat sarcoma virus oncogene homologue; IQR - interquartile range; SD - standard deviation; CEA - carcinoembryonic antigen; N/A - not available*

** Values for some patients are missing.*
