# Supplementary material for: Complete Resection of Simultaneous Hepatic and Pulmonary Metastases from Colorectal Cancer in the Era of Modern Systemic and Surgical Therapies: A Systematic Review and Meta-Analysis
Source: Ann Surg Oncol. 2026 Aug 22;33(12):10065–75. doi: 10.1245/s10434-026-20323-x (PMC13675156; doi:10.1245/s10434-026-20323-x)
Supplement: Supplementary file 6 [file 10434_2026_20323_MOESM6_ESM.docx]

| **Database** | **Search formula** | **Number of records identified** |
| --- | --- | --- |
| Pubmed | (surgery OR resection OR metastasectomy) AND (liver OR hepatic) AND (lung OR pulmonary) AND (metastatic OR metastases OR metastasis) AND (colon cancer OR rectal cancer OR colorectal cancer OR colon neoplasm OR rectal neoplasm OR colorectal neoplasm) AND (mortality OR mortalities OR death OR survival) | 1557 |
| Embase | ('surgery'/exp OR surgery OR 'resection'/exp OR resection OR 'metastasectomy'/exp OR metastasectomy) AND ('liver'/exp OR liver OR hepatic) AND ('lung'/exp OR lung OR pulmonary) AND (metastatic OR 'metastases'/exp OR metastases OR 'metastasis'/exp OR metastasis) AND ('colon cancer'/exp OR 'colon cancer' OR (('colon'/exp OR colon) AND ('cancer'/exp OR cancer)) OR 'rectal cancer'/exp OR 'rectal cancer' OR (rectal AND ('cancer'/exp OR cancer)) OR 'colorectal cancer'/exp OR 'colorectal cancer' OR (colorectal AND ('cancer'/exp OR cancer)) OR 'colon neoplasm'/exp OR 'colon neoplasm' OR (('colon'/exp OR colon) AND ('neoplasm'/exp OR neoplasm)) OR 'rectal neoplasm'/exp OR 'rectal neoplasm' OR (rectal AND ('neoplasm'/exp OR neoplasm)) OR 'colorectal neoplasm'/exp OR 'colorectal neoplasm' OR (colorectal AND ('neoplasm'/exp OR neoplasm))) AND ('mortality'/exp OR mortality OR mortalities OR 'death'/exp OR death OR 'survival'/exp OR survival) AND [01-01-2000]/sd NOT [02-09-2025]/sd | 6015 |
